# Supplementary figures and images for: SenseNet, a tool for analysis of protein structure networks obtained from molecular dynamics simulations
Source: PLoS One. 2022 Mar 17;17(3):e0265194. doi: 10.1371/journal.pone.0265194 (PMC8929561; doi:10.1371/journal.pone.0265194)

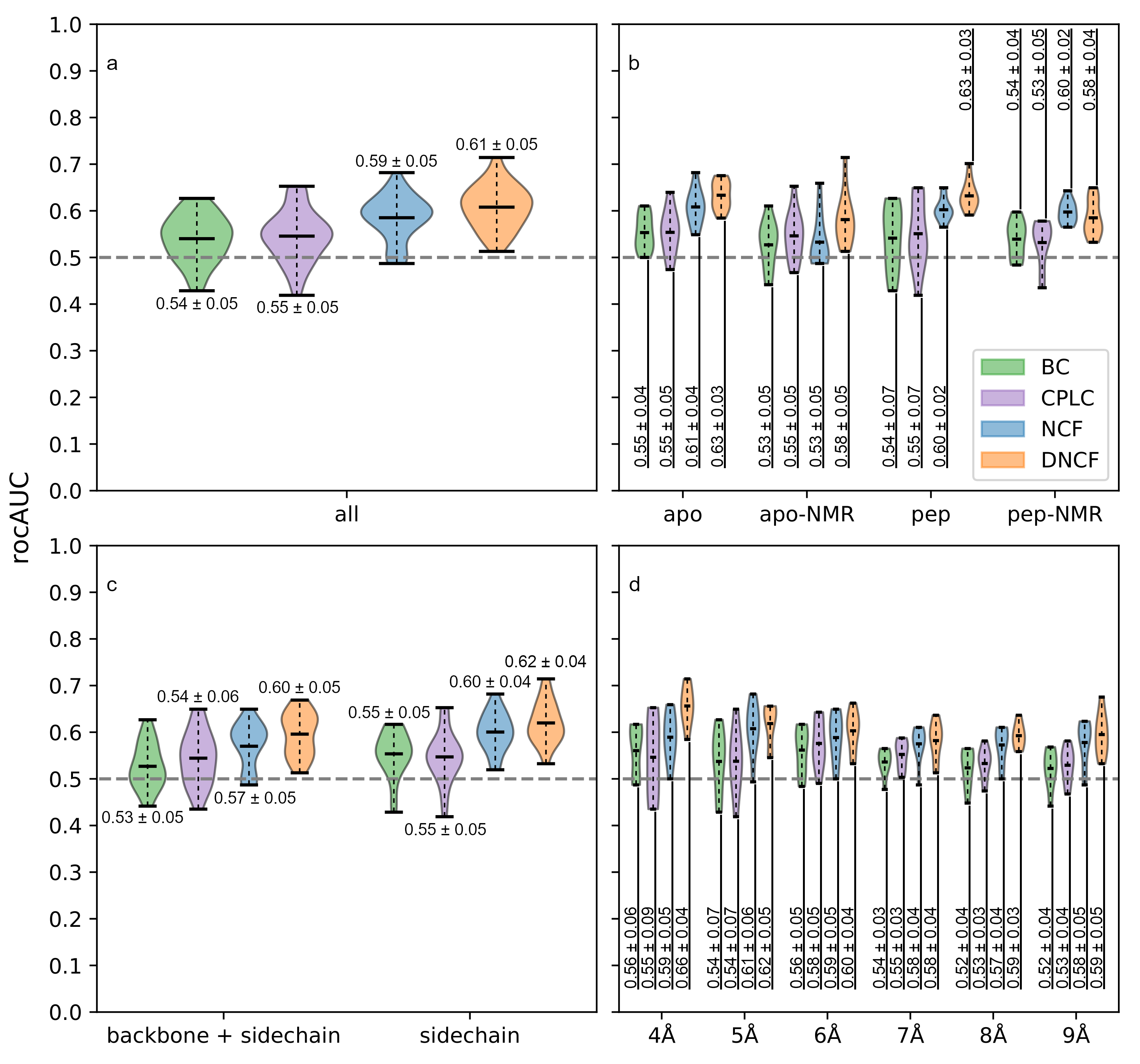

Supplement: S1 Fig — Shaded areas show distribution estimates based on a gaussian kernel with added labels for mean and standard deviation. (a) Distributions including all parameter combinations. (b) Source of analyzed network data: Crystal structures (apo, pep) or NMR based structures (apo-NMR, pep-NMR). (c) Interaction subset: All interactions or sidechain-exclusive networks. (d) Distance cutoff for carbon-carbon contacts in the network. (TIF) [file pone.0265194.s001.tif]
